# Herpes Simplex Virus Pneumonia: Importance of Aspiration Etiology

**DOI:** 10.1155/2019/7623576

**Published:** 2019-12-10

**Authors:** Kentaro Odani, Mitsuhiro Tachibana, Rintaro Tamashima, Yutaka Tsutsumi

**Affiliations:** ^1^Department of General Medicine (Junior Resident), Shimada Municipal Hospital, Shimada, Shizuoka, Japan; ^2^Department of Diagnostic Pathology, Shimada Municipal Hospital, Shimada, Shizuoka, Japan; ^3^Department of Cardiology, Shimada Municipal Hospital, Shimada, Shizuoka, Japan; ^4^Department of Cardiology, Seirei Hamamatsu General Hospital, Hamamatsu, Shizuoka, Japan; ^5^Pathos Tsutsumi, Nagoya, Aichi, Japan

## Abstract

Herpes simplex virus (HSV) pneumonia caused by aspiration from the oropharyngeal exudates is described. An 89-year-old Japanese male in a poor performance state complained of appetite loss followed by difficulty in swallowing, and bilateral pulmonary infiltrates with interstitial reactions were radiologically pointed out. Antibiotics administration was ineffective, and he died on the 6th day of hospitalization. At autopsy, HSV-induced multiple mucosal erosions were observed on the tongue, pharynx, epiglottis, and trachea. In bilateral lower lobes of the lung, HSV infected bronchiolar and type-II alveolar cells in association with acute interstitial reactions. The infected cells with intranuclear inclusion bodies were immunoreactive with HSV antiserum. HSV-1 infection was confirmed by immunostaining with monospecific monoclonal antibodies and by type-specific real-time polymerase chain reaction. It is very likely that HSV pneumonia was provoked by aspiration of infected exudates from the upper airway (namely, sequential infection from the tongue, epiglottis, and trachea to lung). Oropharyngeal herpes might cause anorexia and difficulty in swallowing, probably accelerating aspiration. The medical staff did not recognize the oropharyngeal lesions of this aged patient. We must realize again the importance of oral care for hospitalized patients to avoid aspiration pneumonia, including herpetic pneumonia.

## 1. Introduction

Pneumonia caused by herpes simplex virus (HSV) is seldom seen in immunocompetent patients. HSV-1 commonly causes herpes labialis, gingivostomatitis, and pharyngitis. HSV-1 latently survives in trigeminal and visceral nerve ganglions. An immunocompromised status may provoke reactivation of HSV-1 [1]. Transmission pathways of herpetic pneumonia include (1) aspiration of infected oropharyngeal secretions, (2) continuous downward extension of the infection to the tracheobronchial tree, (3) viral reactivation in vagal nerve ganglia, and (4) systemic dissemination to the lung from a remote site of infection [2]. We experienced an autopsy case of HSV-1 pneumonia in an immunocompetent but old-aged patient. Aspiration etiology of viral infection was considered.

## 2. Case Presentation

The patient was an 89-year-old Japanese male with past history of hypertension, ascending colon adenocarcinoma, superior mesenteric artery dissection, and dementia. He underwent ascending colectomy, while arterial dissection was kept conservatively. He lived in nursing facility by using a wheelchair. He complained of appetite loss for four months, and for the recent two weeks, he could not swallow, so that an intravenous drip infusion started. His activity of daily living became worse, resulting in laying on the bed. Because of dyspnea with coarse crackles and reduction of oxygen saturation (78%), he was hospitalized to the Cardiology department, Shimada Municipal Hospital, Shimada, Shizuoka, Japan. Chest X-ray film showed bilateral pulmonary infiltrates, and CT scan disclosed pulmonary interstitial reactions with a reticular pattern and emphysematous changes. White blood cell count was 9,700/*μ*L and C-reactive protein was 10.75 mg/dL. Arterial gas analyses revealed pH 7.499, PaO_2_ 76.0 mmHg, PaCO_2_ 41.2 mmHg, and anion gap 8.2 mmol/L. Ceftriaxone (2 g/day, i.v.) and then meropenem (1 g/day, i.v.) were administered. Inflammation unchanged, and he died on the 6th day of hospitalization.

**Figure 1:**
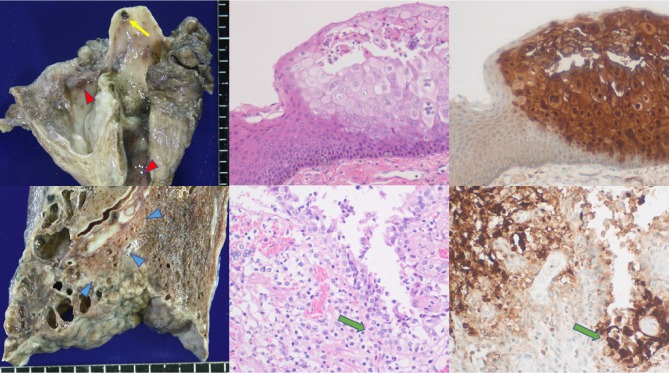
HSV infection in the upper (top panels) and lower (bottom panels) airway. Left: gross appearance after formalin fixation, center: hematoxylin and eosin staining, right: immunostaining for HSV-1 (amino acid polymer method with HSV-1 antiserum). The epiglottis reveals a hemorrhagic ulcer (yellow arrow), while hemorrhagic erosions are distributed on the pharyngeal and tracheal mucosa (red arrowheads). The vesicular mucosal lesion in the pharynx consists of acantholytic squamous cells with intranuclear inclusions and evident immunoreactivity of HSV-1 antigen. The lower lobe of the lung reveals parenchymal infiltration (blue arrowheads), surrounded by subpleural cyst-forming fibrosis. Microscopically, the bronchiolar (green arrows) and parenchymal alveolar cells show intranuclear inclusions with strong HSV-1 immunoreactivity.

At autopsy, multiple mucosal erosions were observed on the tongue, pharynx, epiglottis, and trachea. In bilateral lower lobes of the lung, parenchymal infiltration was seen together with subpleural honeycombing (pulmonary fibrosis). Histologically, foci of HSV infection with acantholysis and intranuclear inclusions were noted on the oropharyngolaryngeal mucosa. HSV was also infected in the lung, and bronchiolar and type-II alveolar cells among acute interstitial reactions contained intranuclear inclusion bodies, strongly suggesting that the viral colonization in the lung parenchyma was mediated by aspiration of the infected secretion from the upper airway. HSV pneumonia was seen in bilateral lower lobes, particularly in the right lower lobe (lung weight: left 230 g, right 470 g). The infected cells with inclusion bodies were strongly immunoreactive for both HSV-1 and HSV-2 antigens, recognized by antisera supplied by Agilent Technologies. Santa Clara, CA, USA (Both antisera were cross-reactive for HSV-1 and HSV-2). HSV-1 infection was confirmed by additional immunostaining with monoclonal antibodies to HSV-1 and HSV-2 [3], as well as by real-time polymerase chain reaction using type-specific primers [4], using formalin-fixed, paraffin-embedded specimens of the tongue and lung. By quantitatively comparing with *β*-actin DNA, the viral loads (HSV-1 copies per cell) were estimated 4.2 in the lung and 1.6 in the tongue. The background lung disclosed cyst-forming septal fibrosis mainly in subpleural region of the lower lobes (seemingly resulting from repeated aspiration pneumonia) and emphysema in the upper lobes.  Figure 1 illustrates representative findings of herpetic infections. No viral infection was observed in the esophagus and stomach. Additional autopsy findings included old anteroseptal myocardial infarction with stenosis of the anterior descending branch of left coronary artery (heart weight 230 g). Severe atherosclerosis was seen in the aorta. No recurrence of colon cancer was noted.

## 3. Discussion

HSV pneumonia usually occurs in immunocompromised patients [5, 6] or in patients with preexisting lung disorders [6, 7]. HSV pneumonia in immunocompetent patients is rare [8, 9]. Our patient was old-aged, and his performance status was poor. The possibility of age-related attenuation in immunity to provoke HSV pneumonia should be considered. In the present case, HSV-1 infection was proven in the tongue, pharynx, epiglottis, trachea, and lung. It is very likely that HSV pneumonia was provoked by aspiration of infected exudates from the upper airway; in other words, the downward sequential infection from the tongue through epiglottis and trachea to lung. The existence of pulmonary fibrosis might have accelerated the viral colonization on the respiratory tree in the present case. Oropharyngeal herpes might cause anorexia and difficulty in swallowing. The patient's anorexia began four months before hospitalization. We suspect that his oral herpetic infection started in this period. Difficulty in swallowing activity could have accelerated aspiration. It is plausible that the fibrotic process gave a chance for the aspirated HSV-1 to colonize the lung parenchyma.

The prognosis of herpetic pneumonia is poor [2], so that the prevention should be of essential importance. Oropharyngeal HSV infection has been confirmed as a risk factor for HSV pneumonia in large cohorts [7, 10]. One of the serious problems in the present case was that the medical staff did not recognize the painful oropharyngeal lesions of the aged patient in a poor performance status. The association of labial herpes was unclear: no description was found in the clinical chart. We learnt from the present case that the medical staff should examine the mouth and tongue of the patient with anorexia. Introduction of routine oral evaluation for the hospitalized patients is definitely needed [11]. Close cooperation by dental and medical staff may lead to early detection of oral infection of HSV to change the drug from antibiotics to acyclovir [12]. We must realize the importance of oral care for all hospitalized patients to avoid aspiration pneumonia, including herpetic pneumonia. In contrast, once herpetic lesions are identified in the oral cavity, the introduction of oral hygiene procedures must be avoided until complete cure of the mucosal lesion, because of the risk of viral transmission to other head and neck areas of the patient or to the staff performing oral care [13].
